# An Outbreak of Carbapenem-Resistant *Klebsiella pneumoniae* in an Intensive Care Unit of a Major Teaching Hospital in Chongqing, China

**DOI:** 10.3389/fcimb.2021.656070

**Published:** 2021-06-02

**Authors:** Lingyi Zeng, Chengru Yang, Jisheng Zhang, Kewang Hu, Jingbo Zou, Jie Li, Jianmin Wang, Wan Huang, Lining Yin, Xiaoli Zhang

**Affiliations:** ^1^ Department of Microbiology, Yongchuan Hospital of Chongqing Medical University, Chongqing, China; ^2^ Department of Microbiology, The First Affiliated Hospital of Jiamusi University, Jiamusi, China; ^3^ Department of Microbiology, Yongchuan District Center for Disease Control and Prevention of Chongqing, Chongqing, China

**Keywords:** intensive care unit, carbapenem-resistant *Klebsiella pneumoniae*, outbreak, molecular epidemiology, ST11

## Abstract

**Background:**

Due to the critical condition and poor immunity of patients, the intensive care unit (ICU) has always been the main hospital source of multidrug-resistant bacteria. In recent years, with the large-scale use of antibiotics, the detection rate and mortality of carbapenem-resistant *Klebsiella pneumoniae* (CRKP) have gradually increased. This study explores the molecular characteristics and prevalence of CRKP isolated from the ICU ward of a tertiary hospital in China.

**Methods:**

A total of 51 non-duplicated CRKP samples isolated from the ICU were collected from July 2018–July 2020. The enzyme production of the strains was preliminarily screened by carbapenemase phenotypic test, and drug-resistant and virulence genes were detected by PCR. The transferability of plasmid was verified by conjugation test. The minimal inhibitory concentration (MIC) was determined by microbroth dilution method and genetic diversity was detected by multilocus sequence typing and pulsed-field gel electrophoresis.

**Results:**

*bla_KPC-2_* was the only carbapenemase detected. The major virulence genes were *uge* (100%), *mrkD* (94.1%), *kpn* (94.1%), and *fim-H* (72.5%), while *wcag*, *ironB*, *alls* and *magA* genes were not detected. One sequence type ST1373 strain, hypervirulent *K. pneumoniae* (hvKP), was detected. CRKP strains were highly resistant to quinolones, cephalosporins, aminoglycosides, and polymyxin, but susceptive to tigecycline and ceftazidime–avibactam. The success rate of conjugation was 12.2%, indicating the horizontal transfer of *bla_KPC-2_*. Homology analysis showed that there was a clonal transmission of ST11 CRKP in the ICU of our hospital.

**Conclusion:**

The present study showed the outbreak and dissemination in ICU were caused by ST11 CRKP, which were *KPC-2* producers, and simultaneously, also carried some virulence genes. ST11 CRKP persisted in the ward for a long time and spread among different areas. Due to the widespread dispersal of the transferable *bla_KPC-2_* plasmid, the hospital should promptly adopt effective surveillance and strict infection control strategies to prevent the further spread of CRKP. Ceftazidime–avibactam showed high effectiveness against CRKP and could be used for the treatment of ICU infections.

## Introduction

In clinical work, *Klebsiella pneumoniae* (*K. pneumoniae*) is a common pathogen that can cause multiple infections of wounds, the respiratory tract, urinary tract, and pleural effusion (Podschun and Ullmann, 1998; Holt et al., 2015; Lee et al., 2017; Moradigaravand et al., 2017). In recent years, *K. pneumoniae* has been identified as a major cause of hospital-acquired pneumonia and is responsible for approximately 10% of all hospital-acquired infections, ranking second among Gram-negative pathogens (Abdelsalam et al., 2018). With the increasing overuse of common antibiotics, carbapenem-resistant *K. pneumoniae* (CRKP) strains have spread worldwide. CRKP strains can produce a variety of carbapenemases and/or extended spectrum β lactamases (ESBLs) combined with loss of membrane porins and overexpression of efflux pumps, eventually resulting in multidrug-resistant (MDR), extensively drug-resistant (XDR), and pandrug-resistant (PDR) bacteria (Magiorakos et al., 2012; Bhatt et al., 2020). Since the mid-1980s, hypervirulent *K. pneumoniae*(hvKP) has emerged as a clinically significant pathogen responsible for serious disseminated infections. A variety of hypervirulence-associated factors are important in hvKP strains, including capsular serotypes, a pathogenicity island and several virulence factors (Lee et al., 2017). *rmpA* activates capsule production, resulting in the hypermucoviscosity phenotype and increase in virulence (Cheng et al., 2010). *aero *and *iroNB *which are important siderophores play a crucial role in the progression of infection; *magA *and *wcaG* are associated with capsule serotype. Bacteria colonization and fimbriae adhesion are mainly related to *fimH*, *mrkD*, *allS*, and *kpn*; and *uge* affects the expression of bacterial lipopolysaccharide. The acquisition of resistance and virulence has increased the mortality rate of CRKP (Chen et al., 2020).

The hospital intensive care unit (ICU) treats and rescues critically ill patients and provides precise treatment and careful nursing intervention, which plays an important role in clinical practice. Due to serious illnesses, complicated etiology, and low resistance of ICU patients, the infection and mortality rates are significantly higher than other departments, and has become a significant factor affecting the prognosis of patients. The risk of CRKP infection in ICU patients is amplified due to the use of antibiotics and long-term hospitalization (Friedman et al., 2017; GiVi et al., 2018).Extremely resistant or (and) highly virulent CRKP infections are often complicated, difficult to treat, and have high mortality. In addition, due to limited space in the ICU, cross-spreading and even outbreaks are extremely common, while patients have poor immunity (Tian et al., 2016; Gu et al., 2018).

The purposes of this study was to elucidate the patterns of antibiotic resistance of infectious CRKP strains, determine resistance and virulence genes frequencies, and investigate the genetic diversity of CRKP isolated from the ICU. We aim to provide molecular and epidemiological data to aid in prevention of the future emergence and outbreak of drug-resistant bacteria.

## Materials and Methods

### Bacterial Collection

The Yongchuan Affiliated Hospital of Chongqing Medical University is a leading teaching hospital in Chongqing, a city in Chongqing province, China. The hospital has 1,480 beds in 47 wards. It is one of the largest health care centers in the west Chongqing province, and is responsible for the medical care of a population estimated at 10 million. A total of 51 non-duplicated clinical CRKP isolates were collected from the ICU from July 2018–July 2020. Patient information including age, gender, length of ICU admission, diagnosis, and outcome was obtained from electronic medical records. All isolates were obtained from various clinical specimens and identified by a VITEK-2 automated microbiology analyzer (bioMérieux, France). Following the breakpoints of the Clinical and Laboratory Standards Institute (CLSI-2020) guidelines (CLSI, 2020), clinical isolates that are not susceptible to carbapenems (imipenem, meropenem, or ertapenem) were used as experimental subjects. All isolates were stored at −80°C for further study.

### Antimicrobial Susceptibility Testing

The VITEK-2 Compact automatic microbiological analyzer AST-GN card (bioMérieux, France) was used for routine antimicrobial susceptibility testing. Minimum inhibitory concentration (MIC) defined as the lowest compound concentration (µg/ml) required to stop bacterial growth was determined by using the microbroth dilution method. Imipenem (IPM), meropenem (MEM), amikacin (AK), levofloxacin (LEV), tigecycline (TIG), polymyxin B (PB), and ceftazidime–avibactam (CAZ–AVI) were used to determine the MIC by the microbroth dilution method. ATCC 25922, ATCC 700603, and BAA-1705 were used as quality control strains. Three parallel assays were performed for each sample. The IPM, MEM, AK, LEV, PB and CAZ-AVI results were interpreted based on CLSI criteria (CLSI, 2020), whereas the TIG results were interpreted based on the European Committee on Antimicrobial Susceptibility Testing (EUCAST) (The European Committee on Antimicrobial Susceptibility Testing, 2020) breakpoint recommendations.

### String Test

Following reference (Zhang et al., 2016),CRKP strains were incubated overnight on blood agar. A single colony was touched with a loop and stretched outward. The length of the viscous string was pulled upward and measured. A positive string test result was defined as a string longer than 5 mm. The string test was repeated three times for each.

### Carbapenemase Phenotypic Test

The modified carbapenem inactivation method (mCIM) and EDTA-modified carbapenem inactivation method (eCIM) were simultaneously performed to detect carbapenemase production in CRKP. Briefly, 2 ml aliquots of trypticase soy broth (TSB) were directly inoculated with a 1 μl loopful of CRKP colonies. The suspension was vortexed and a 10 μg meropenem disk (Oxoid) was placed in the inoculated tube. Tubes were incubated for 4 h (± 15 min) and placed on a Mueller–Hinton agar (MHA) plate after lawn inoculation of 0.5 McFarland bacterial suspension of meropenem-susceptible *E. coli* ATCC 25922. For eCIM testing, each isolate was inoculated in a 2 ml aliquot of TSB-EDTA (EDTA concentration, 5 mM). Isolates were incubated and plated as described for mCIM testing. Plates were incubated for 18 to 24 h, and the interpretation for both assays was according to CLSI-2020 (CLSI, 2020).

### Molecular Detection of Resistance and Virulence Genes

DNA was extracted from each strain of CRKP by the boiling method (Gong et al., 2018; Unlu and Demirci, 2020). Resistance genes were detected by polymerase chain reaction (PCR), including carbapenemase genes (*bla_KPC_*, *bla_NDM_*, *bla_IMP-4_*, *bla_IMP-8_*, *bla_VIM-1_*, *bla_VIM-2_*, and *bla_OXA-48_*), ESBL genes (*bla_SHV_*, *bla_TEM_*, *bla_CTX-M-1_*, and *bla_CTX-M-9_*), AmpC β-lactamase enzymes (*bla_DHA_* and *bla_ACC_*), and quinolone resistance genes (*qnrA*, *qnrB*, *qnrS*, *qepA *and *aac(6’)Ib-cr*).Virulence genes detected include: *fim-H*, *magA*, *aero*, *alls*, *iroNB*, *kpn*, *mrkD*, *rmpA*, *uge* and *wcaG*. All primers refer to previous studies (Gay et al., 2006; Park et al., 2006; Ma et al., 2009; El Fertas-Aissani et al., 2013; Compain et al., 2014; Wasfi et al., 2016; Jian-Li et al., 2017; Fu et al., 2018; Gong et al., 2018). Positive amplification products were sequenced, and the sequencing results were compared using Basic Local Alignment Search Tool (BLAST), available at https://blast.ncbi.nlm.nih.gov/Blast.cgi.

### Conjugation Experiment

To assess whether the carbapenemase-producing genes were located on plasmids and to assess the transferability of these genes, strains underwent conjugation with *E. coli EC600*. The conjugation experiment was carried out using a membrane bonding experiment as previously described (Gong et al., 2018). Both the donor (CRKP) and the recipient strains (*E. coli EC600*) were mixed in Luria–Bertani broth at a ratio of 1:3, and the mixtures were placed on a membrane and incubated for 24 h at 35°C. Transconjugants were selected on MHA plates supplemented with rifampicin (600 μg/ml) and meropenem (1 μg/ml). Colonies that grew on the selective medium were identified by the VITEK-2 Compact system and *16S rRNA* sequence. Strains that harbored carbapenemase and exhibited higher MICs of resistance to carbapenems than *EC600* were defined as the transconjugants and the presence of resistance determinants was confirmed by PCR.

### Multilocus Sequence Typing (MLST)

MLST was performed using seven housekeeping genes of *K. pneumoniae* that were amplified using primers found in online databases (http://bigsdb.pasteur.fr/klebsiella/primers_used.html). PCR products were sequenced, and sequence types (STs) were determined using online database tools (https://bigsdb.pasteur.fr/klebsiella/klebsiella.html).

### Pulsed-Field Gel Electrophoresis (PFGE)

Genomic DNA from CRKP strains was prepared in agarose plugs and digested with the restriction enzyme Xba*1* for 3 h at 37°C. The digested fragments were separated on a 1% pulsed-field certified agarose using the Bio-Rad CHEF Mapper System under the following conditions: temperature of 14°C, voltage of 6 V/cm, run time of 18 h, and a switch time of 5–35 s. PFGE patterns were identified according to the protocol at the Centers for Disease Control and Prevention (CDC) website, and the band patterns were analyzed using BioNumerics Software. The analysis was conducted using the unweighted pair group method and the arithmetic mean (UPGMA) using a dice coefficient to judge the strain’s affinity, clusters were defined as DNA patterns sharing ≥80% similarity.

## Results

### Clinical Characteristics

**Figure 1:**
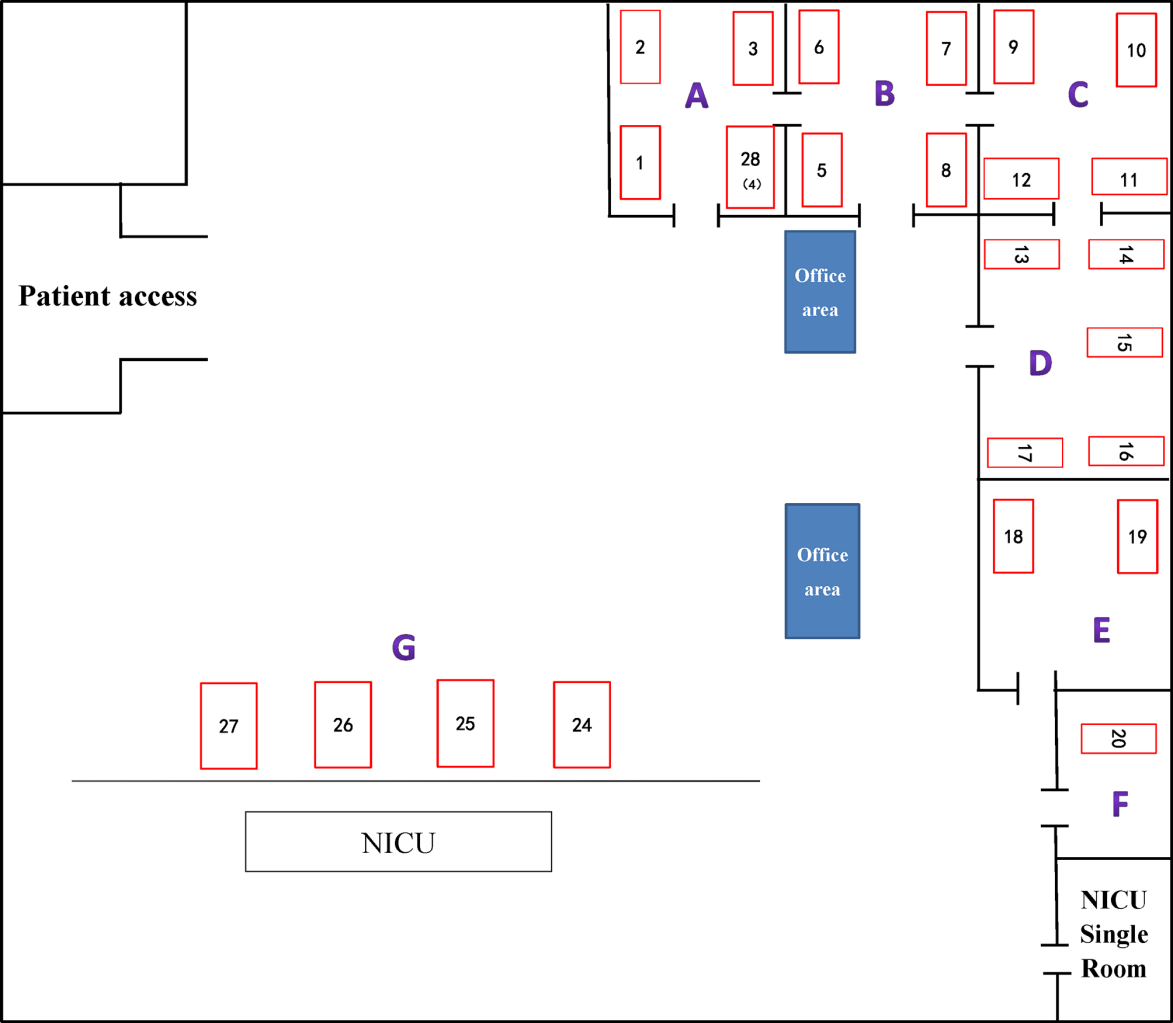
ICU internal layout plan. The number represents the corresponding beds. **(A–G)** indicate different areas divided artificially.

Among 51 unique isolates, 31 (60.8%) strains were obtained from sputum, 13 (25.5%) from lung lavage fluid, four (7.8%) from urine, two (3.9%) from blood and one from secretion. Most patients had pulmonary disease and received invasive surgical treatments, such as endotracheal intubation, or invasive ventilation. Mortality and improvement rates of patients after infection were similar, 13.7% VS 17.6%, respectively. According to a diagram of the ICU layout, groups A and D were the areas with the highest detection rate (**Figure 1** and **Table 1**).

**Table 1:** Clinical characteristics of ICU patients.

Clinical characteristic	N = (51)
**Average age**	72 years (43–94years)
**Male gender**	41 (82%)
**Origin**	
A group	13 (25.5%)
B group	6 (11.8%)
C group	8 (15.7%)
D group	13 (25.5%)
E group	5 (9.8%)
F group	0 (0%)
G group	6 (11.8%)
**Comorbidities**	
Diabetes mellitus	4 (7.8%)
Pulmonary disease	45 (88.2)
Invasive operation	44 (86.3)
Antibiotic exposure	35 (68.6)
**Outcomes**	
Improvement	7 (13.7%)
Mortality	9 (17.6%)
Abandon treatment	8 (15.7%)
Discharge request	27 (52.9%)

### Results of Antimicrobial Susceptibility Testing

**Figure 2:**
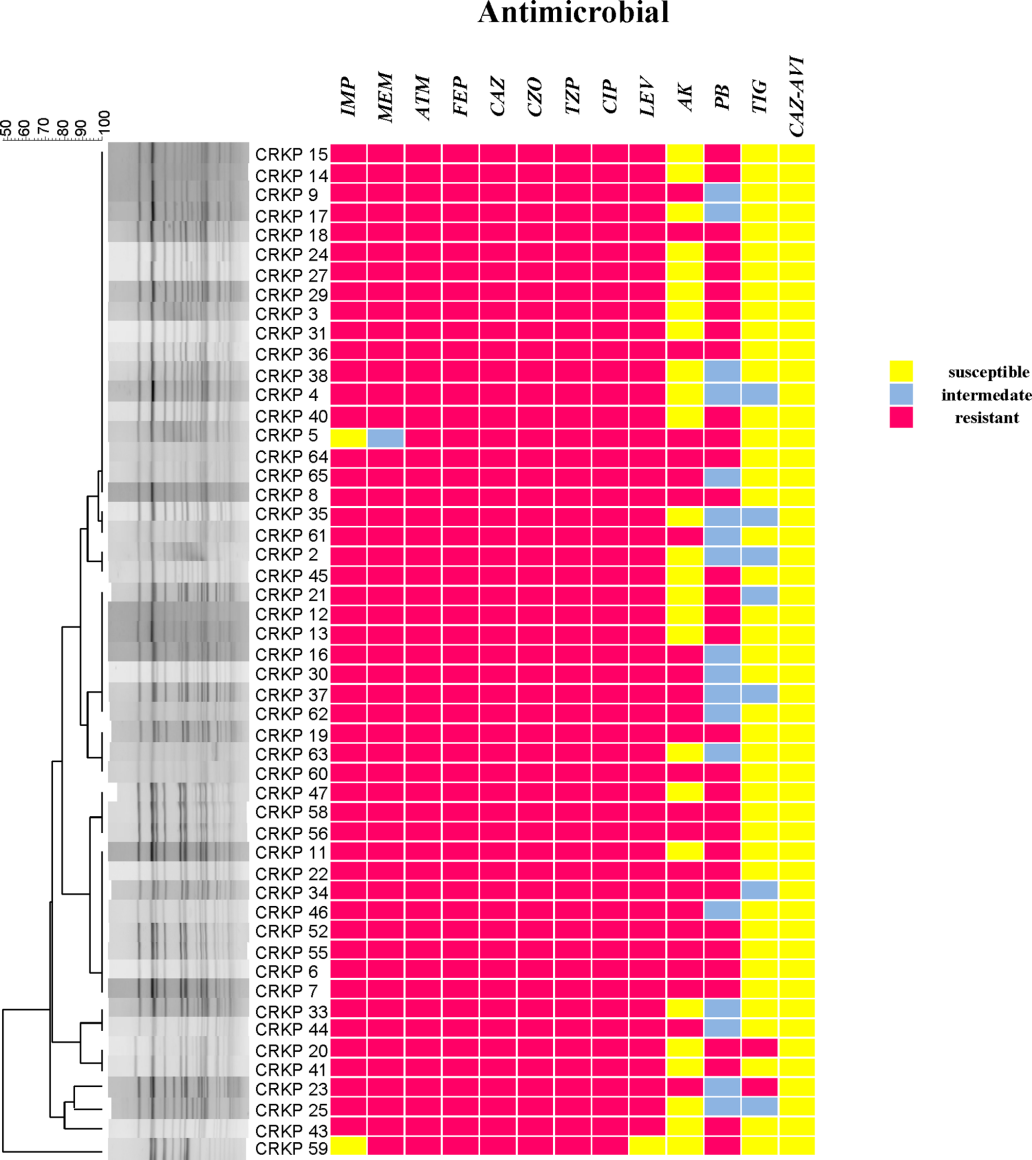
CRKP genetic similarity and antibiotic sensitivity. IMP, Imipenem; MEM, Meropenem; ATM, Aztreonam; FEP, Cefepime; CAZ, Ceftazidime; CZO, Cefazolin; TZP, Piperacillin tazobactam; CIP, Ciprofloxacin; LEV, Levofloxacin; AK, Amikacin; PB, Polymyxin B; TIG, Tigecycline; CAZ–AVI, ceftazidime–avibactam.

**Figure 3:**
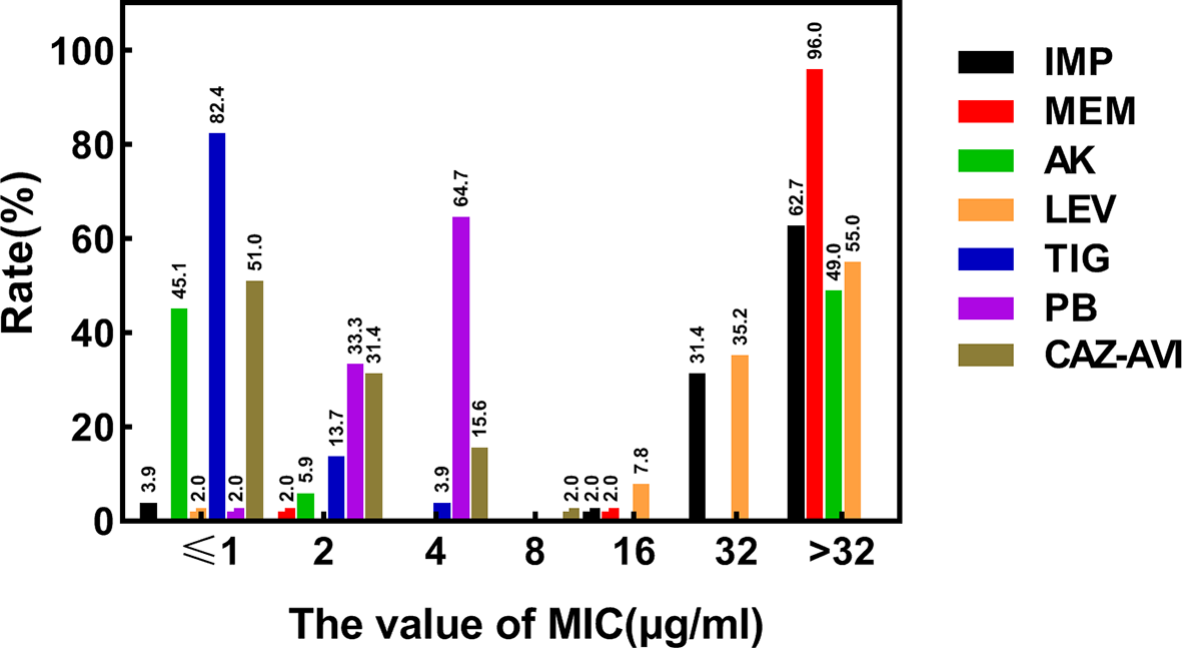
Distribution of MIC values of different types of antibiotics determined by broth micro-dilution method. MIC, the minimal inhibitory concentration; µg/ml, micrograms per milliliter; IMP, Imipenem; MEM, Meropenem; LEV, Levofloxacin; AK, Amikacin; PB, Polymyxin B; TIG, Tigecycline; CAZ–AVI, ceftazidime–avibactam.

According to the results of the susceptibility testing, all strains were defined as MDR (means ‘resistant to three or more antimicrobial classes’) (Magiorakos et al., 2012). In addition to being resistant to carbapenem antibiotics, all CRKPs had high resistance to quinolones, cephalosporins, and monocyclics. The drug resistance rate to amikacin was 49.0%, and that of polymyxin B was 64.7%. Two strains (3.9%) showed resistance to tigecycline, while seven strains (13.7%) expressed intermediate susceptibility. All strains were susceptible to ceftazidime–avibactam (**Figure 2**). Various antibiotics showed different ranges of MIC values, and the specific MIC value distribution is shown in **Figure 3**.

### String Test

Only four out of 51 CRKP isolates (7.8%) exhibited the hypermucoviscous phenotype during the string test, the rest were negative.

### Carbapenemase Phenotypic Experiment

**Figure 4:**
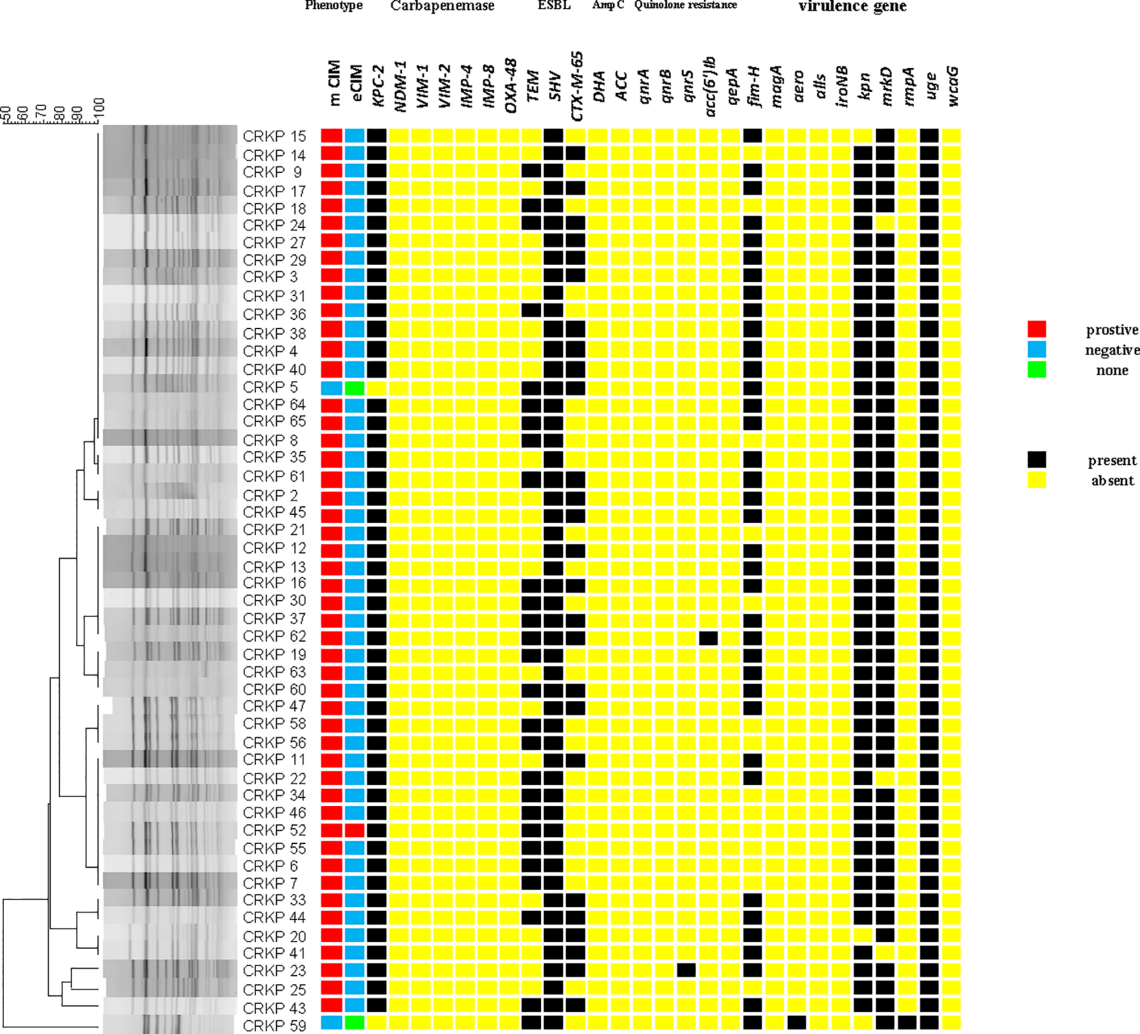
The carrying status of phenotype, drug resistance genes, virulence genes and homology heat map of CRKP.

The positive rate of the mCIM test was 96.0% (49/51), and one (2.0%) CRKP strain was positive for eCIM. This result shows that in the ICU of this hospital, serine carbapenemases are mainly present (**Figure 4**).

### Detection of Resistance and Virulence Genes

*bla_KPC-2_* (96.1%) was the only carbapenem resistance gene detected. This is consistent with the results of carbapenemase phenotypic experiments. *bla_SHV_*(100%), *bla_TEM_* (52.9%), and *bla_CTX-M-65_* (51.0%) had high detection rates. *bla_CTX-M-65_* is a common allelic variant of *bla_CTX-M-9_*, and there are three mutations between them including: A80V; A234V and S275R. AmpC β-lactamases were not detected. Only *qnrS* (2.0%) and *aac(6’)Ib-cr* (2.0%) were detected in plasmid-mediated quinolone resistance genes (PMQR). For virulence genes, *uge* (100%), *mrkD* (94.1%), *kpn* (94.1%), and *fim-H* (72.5%) had different detection rates. Both *aero* (2.0%) and *rmpA* (2.0%) were detected in only one isolate. *wcaG*, *iroNB*, *alls*, and *magA* were not detected (**Figure 4**).

### Conjugation Experiment

There were six strains of *E. coli* identified as conjugants by VITEK-2 and *16S rRNA*. The conjugation success rate was 12.2% (6/49: For strains without *bla_KPC_* production, no conjugation experiment was carried out). All transconjugants showed resistance to both meropenem and rifampicin, and the successful transfer of the *bla_KPC-2_* gene was confirmed by PCR. The other resistance genes carried by the transconjugants are shown in **Table 2**. Compared with the original donor, the MIC value of the transconjugants for carbapenems (IMP, MEM) was significantly decreased (4–64 times), similarly, LEV and PB were also decreased. On the contrary, AK, TIG, and CAZ-AVI did not show obvious changes, except for CRKP*J55*, which had a change in CAZ-AVI. The specific information on the donor and transconjugants susceptibility profiles is shown in **Table 2**.

**Table 2:** Antibiotic susceptibilities of CRKP isolates and their transconjugants (µg/ml).

Isolate	Resistance genes	MIC						
		IMP	MEM	AK	LEV	TIG	PB	CAZ–AVI
CRKP isolates								
*CRKP30*	*KPC-2 TEM SHV*	16	128	>512	32	<1/2	1	1,4
*CRKP 46*	*KPC-2 TEM SHV*	64	128	>512	64	1	2	1,4
*CRKP 55*	*KPC-2 TEM SHV*	64	512	>512	32	<1/2	4	4,4
*CRKP 56*	*KPC-2 TEM SHV*	128	512	>512	32	<1/2	4	1,4
*CRKP 58*	*KPC-2 TEM SHV*	64	512	>512	32	1	4	4,4
*CRKP 63*	*KPC-2 SHV*	256	256	<1/2	32	1	2	4,4
*E. col* transconjugant strains								
*CRKPJ30*	*KPC-2 TEM*	4	4	>512	<1/2	1	<1/2	1/2,4
*CRKPJ46*	*KPC-2 TEM*	4	4	>512	<1/2	<1/2	<1/2	1/2,4
*CRKPJ55*	*KPC-2 TEM*	4	16	>512	1	<1/2	1	>256,4
*CRKPJ56*	*KPC-2 TEM*	8	8	>512	<1/2	<1/2	<1/2	1,4
*CRKPJ58*	*KPC-2 TEM*	16	16	>512	<1/2	<1/2	<1/2	1,4
*CRKPJ63*	*KPC-2*	4	4	<1/2	1	1	<1/2	1/2,4

IMP, imipenem; MEM, meropenem; AK, amikacin; LEV, levofloxacin; TIG, tigecycline; PB, polymyxin B; CAZ–AVI, ceftazidime–avibactam; µg/ml, micrograms per milliliter.

### Homology Comparison

Two sequence types (STs) were identified among all isolates, with 50 (98.0%) belonging to ST11, and one (2.0%) being an isolate of ST1373.The PFGE results were interpreted according to international criteria and allocated into clusters using a cut-off value of 80% genetic similarity, all isolates were divided into five different clone coincidence clusters (A–E). Cluster A was composed of 32 (62.7%) ST11 isolates, representing the largest group, while 11 (21.6%) isolates belonged to cluster B, four (7.8%) isolates and three (5.9%) isolates were from clusters C and D, respectively. Cluster E (2.0%) contained only one isolate, which belonged to ST1373. Both detection methods showed high homology of CRKP in the ICU (**Figures 2**, **4**).

## Discussion

Many studies have reported *K. pneumoniae* as the most common infectious organism in the ICU (Liu et al., 2016; Xu et al., 2017). Our study reported an outbreak of ST11 carbapenem-resistant *K. pneumoniae* in the ICU of a teaching hospital. Several important points can be made from the data gathered from this ICU outbreak.

**Figure 5:**
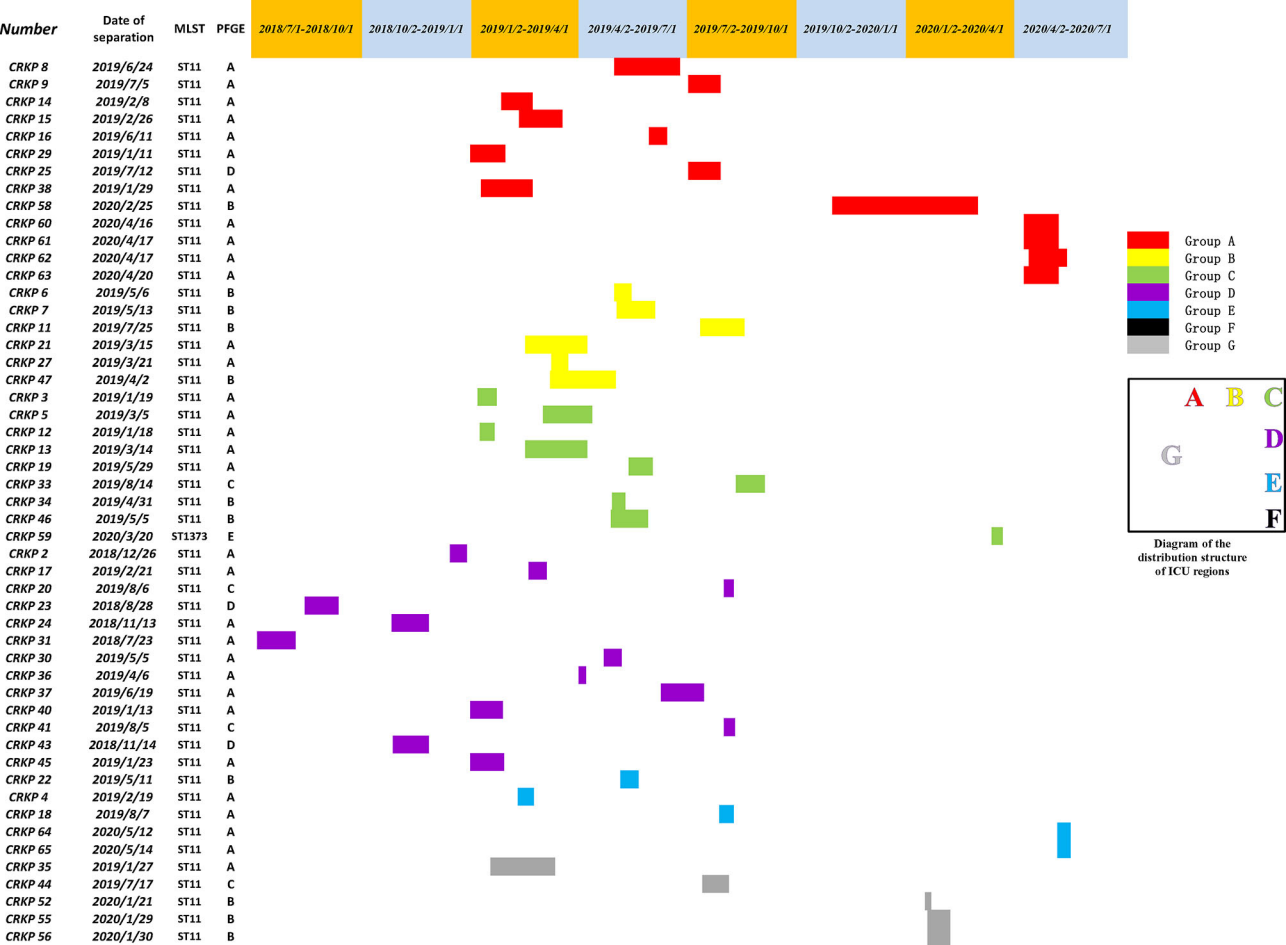
A timeline represents the ICU stay of patients. Different colors in the legend indicate the different groups.

First, combining the timeline and spatial location distribution of this ward, we found that the emergence of drug-resistant bacteria first occurred in group D, and gradually spread to adjacent groups, and staying in group A for the longest time. Groups A and D have the highest detection rate, and group F had no detection (may be related to the small number of beds in this area). The outbreak timeframe was concentrated from January 2019 to October 2019. Due to the impact of COVID-2019, the number of patients in our hospital decreased from January 2020, and was mainly concentrated in group A (**Figure 5**). Although all samples were divided into five clusters by PFGE, homology of four of the clusters was very high, excluding cluster E (**Figure 4**). This shows that the outbreak could be traced to the horizontal spread of a certain strain in different wards, which is most likely from nosocomial infection. MLST showed that all strains of clusters A–D belonged to the ST11 type, which is also the most prevalent CRKP type in China. ST11 has recently been reported elsewhere in China, causing fatal infections and high mortality in other hospitals (Zhou et al., 2015; Hu et al., 2016; Sui et al., 2018).

Second, most ST11 CRKP isolates (49/50, 98.0%) in this outbreak produced *bla_KPC-2_* carbapenemase which has been reported to be the most widely spread carbapenemase in China (Qi et al., 2011). The positive rate of the mCIM test was 96.1% (49/51), while the positive rate of eCIM, based on mCIM, was only 2.0% (1/49). This indicates that the main CRKP enzyme type in the ICU of our hospital was serine carbapenemase, not metallo-β-lactamase (MBL). Interestingly, for the single eCIM-positive strain, CRKP52, no MBLs within the coverage of the primers used in this study were detected by PCR, so we speculated that the bacteria may produce other allelic variants out of range or uncommon-MBLs (for example: *bla_SIM_*, *bla_GIM_* or *bla_AIM_*), indicating that the high accuracy and sensitivity of phenotyping experiments, mCIM and eCIM, can be widely applied, and would be useful in departments that require active inspection. By PCR detection, we found that 98.0 and 100% ST11 CRKP harbored *bla_KPC−2_* and *bla_SHV_*, respectively, and most were also positive for *bla_TEM_* and *bla_CTX−M-65_*, consistent with previous reports (van Dorp et al., 2019). This indicates that increasingly serious threats by ST11 are emerging. *bla_KPC-2_* was responsible for the observed resistance in most of the CRKP isolates in the ICU, and is consistent with other reports (Shen et al., 2009; Hu et al., 2020), a typical plasmid-mediated drug resistance gene, that is widely distributed in different sizes and types of plasmids (Jain et al., 2013; Pitout et al., 2015). The success of the conjugation experiment in this study demonstrated the transferability of drug-resistant plasmids. The acquisition of conjugant resistance also showed that drug resistance was transferable. *bla_TEM_* is often part of the genetic environment of *bla_KPC-2_* (Liu et al., 2021; Zhai et al., 2021), so both genes were transferred within the plasmid. In Europe and the United States, transposon Tn*4401* is the most common gene element carrying *bla_KPC_* (Naas et al., 2008; Wang et al., 2015). Tn*1721* is the most widespread transposon containing *bla_KPC-2_* in China, and often appears in the structure of *IRR-tnpA-tnpR-IRL1-IRL2* (Jiang et al., 2010). The horizontal transmission of drug-resistant plasmids can accelerate the diffusion of multidrug-resistant genes and mediate the production of multidrug-resistant strains. The results of conjugation showed that the MIC value of conjugants for carbapenems decreased, indicating that other causes of drug resistance, such as membrane protein, or efflux pump, for example, did not transfer with the plasmid. *bla_KPC_*-resistant plasmids can encode a variety of virulence and retention factors at the same time, which significantly improves the adaptability of *bla_KPC_* to the external environment (Adler et al., 2012), conducive to its global spread. These issues make horizontal transmission research an area of great importance in order to avoid outbreaks.

In addition to carbapenems, we also observed high resistance to aminoglycosides (49.0%) and quinolones (100%) in ICU patients. According to **Table 2**, in addition to the donor bacteria, the conjugants also showed high resistance to aminoglycosides, which indicated that this resistance was mainly mediated by plasmids. Aminoglycoside-related resistance genes: *aph(3’)-Ia*; *aph(3’’)-Ib* and *aph(6)-Id*, etc., were transferred together with the plasmid, resulting in the persistence of high aminoglycoside resistance. Interestingly, we did not detect a high rate of PMQR (*qnrS*: 2.0%, *aac(6’)Ib-cr*: 2.0%). We suspect that this may be related to chromosome mediated quinolone resistance-determining regions (QRDR) (Zeng et al., 2020). These CRKP may have mutations at *gyrA*, *parC*, and other sites, and the susceptibility of conjugants to quinolones also confirmed our assumption. Simultaneously, we also observed a high rate (64.7%) of polymyxin resistance (PR). Polymyxins (including colistin and polymyxin B) are considered antimicrobials of last resort for the treatment of carbapenem-resistant Enterobacteriaceae (CRE) infections (Macesic et al., 2020). Despite their toxicity, they have been increasingly used in the last decade, leading to concern about the emergence of PR (Poirel et al., 2017). Exposure to polymyxins is considered a common risk factor (Richter et al., 2018); however, in our study, only one patient was treated with polymyxin B, and the *mcr-1* gene was not detected. Therefore, we tested the *PmrA/PmrB* and *PhoP/PhoQ* two-component systems and the *mgrB* gene (a regulator of the *PhoP/PhoQ* system), which leads to polymyxin resistance by modification of the lipopolysaccharide target (Aires et al., 2016). There were different types of deleterious mutations in *PhoP/PhoQ* and *PmrB*, including: *PhoP:*I201F, *PhoQ*:D150G, *PmrB:*N8T, T228A, R256G, and L254F. All the PR strains exhibited alterations in the *mgrB* gene, including disruption by IS*5*, IS*3*, and IS*1343*. Similar results have been reported in other studies (Cannatelli et al., 2014; Cheng et al., 2015; Wright et al., 2015; Aires et al., 2016; Pitt et al., 2018). PR could not be transferred to the conjugating receptor with the movement of plasmid, and confirmed that PR was chromosomal. There were reports that the use of third generation cephalosporins, quinolones, and carbapenems within 30 days of infection by *K. pneumoniae* increased the risk of CRKP infection by 2.02, 1.76, and 2.67 times, respectively (Li et al., 2019). Rational and effective use of antibiotics is one of the important means to prevent infection, so close attention must be paid to the signs of antibiotic resistance.

Third, the detection rate of hvKP in this ICU was not as high as previously reported (Zhan et al., 2017). Hypermuscoviscousity and hypervirulence are not always detected together, and the string test is not sensitive enough to detect the virulence (Yang et al., 2020), so following Yu et al. (2017), we define hvKP as a strain that is positive for both the string test and the *rmpA* gene at the same time. In addition, aerobactin has been considered to be an important virulence determinant for hvKP (Zhang et al., 2016). The only hvKP detected in this study carried both *rmpA* and *aero* on the basis of a positive string test. However, the ST1373 hvKP we identified does not produce *bla_KPC_* like other CRKPs, and maintains susceptibility to several drugs such as IMP, LEV and AK, which may be due to the small number of drug resistance genes it carries. This is fortunate, and shows that there is an alternative treatment for the hvKP found in our hospital. Unfortunately, because the patient stopped treatment and no valid contact information is available, we cannot continue follow up. However, the high detection rate of other virulence genes, such as *fimH*, *mrkD*, and *kpn*, related to bacterial colonization and fimbriae adhesion and *uge*, related to bacterial lipopolysaccharide, indicates potential virulence risk. Clinicians in our hospital should pay close attention to the trend of hvKP resistance.

Finally, we have found that some drugs and measures can be used as a potential way to treat and prevent CRKP infection. All strains were susceptible to CAZ–AVI. Ceftazidime/avibactam is a novel β-lactam (ceftazidime) and β-lactamase inhibitor (avibactam) combination that inactivates the active site of serine-β-lactamases, including Ambler class A extended-spectrum β-lactamases (ESBLs), Ambler class C AmpC β-lactamases, the class A carbapenemases, and some class D carbapenemases (van Duin and Bonomo, 2016; Kazmierczak et al., 2018). CAZ–AVI represents a promising therapeutic option to combat KPC-producing *K. pneumoniae.* However, the increased reporting of resistance, with or without prior exposure to the compound, is a matter of concern (Venditti et al., 2019), therefore, the development of novel antibiotics or the combination therapy of two or more antibiotics instead of monotherapy in CRKP-infected patients are feasible options that will effectively delay the production of drug-resistant bacteria and increase their cure rate. The only CAZ-AVI resistant strain we identified was CRKP*J55*, and the original donor did not show resistance. We speculate that the reason for this phenomenon may be related to the difference of transcriptome expression under antibiotic pressure. This requires further in-depth research. We also found 88.2% of the patients suffered from pulmonary disease and most of the samples came from sputum. Because breathing was often obstructed, more than half of the patients received invasive operations such as endotracheal intubation and tracheotomy. Yi et al. have reported that invasive mechanical ventilation for ≥48 h and parenteral nutrition for ≥48 h were risk factors for CRKP infection (Li et al., 2019). Therefore, unnecessary interventional apparatus in the ICU should be removed as early as possible to prevent nosocomial-acquired infections, and enteral feeding should be established as soon as possible to reduce risk factors.

In conclusion, our study described an outbreak of ST11 CRKP that exhibited virulence and long-term persistence in the ICU of a comprehensive hospital in China. The outbreak had multi-drug resistant bacteria that mainly produced *bla_KPC-2_* and could be transferred horizontally. Although this study did not conduct long-term and systematic sampling of all potentially contaminated areas in the ICU, there is a need for understanding the dynamic distribution and spread of CRKP, a major threat to clinical treatment that cannot be ignored. Timely and effective infection control measures are essential to contain and mitigate the risk of nosocomial transmission and outbreaks in hospitals. According to the European Society of Clinical Microbiology and Infectious Diseases (ESCMID) guidelines, the implementation of hand hygiene education programs, contact precautions, and use of alert codes to promptly identify patients with CRKP infections should be applied and infected patients should be isolated. In addition, a program of active screening culture, and implementation of an antimicrobial stewardship program, should be implemented to reduce transmission of multidrug-resistant Gram-negative bacteria in hospital patients, especially in ICUs (Tacconelli et al., 2014; Tiri et al., 2020).

## Data Availability Statement

The original contributions presented in the study are included in the article/supplementary material. Further inquiries can be directed to the corresponding author.

## Author Contributions

XZ conceived of and designed the study. LZ and CY wrote this paper and contributed equally to this work. JSZ, JW, KH, and WH performed the experiments. JL, JBZ, LY, and LZ analyzed the data. All authors contributed to the article and approved the submitted version.

## Funding

This work was supported by General projects of Chongqing Natural Science Foundation (cstc2020jcyj-msxm0067) and Talent introduction project of Yongchuan Hospital of Chongqing Medical University (YJYJ202005, YJYJ202004).

## Conflict of Interest

The authors declare that the research was conducted in the absence of any commercial or financial relationships that could be construed as a potential conflict of interest.
